# Improvement of Single Event Transient Effects for a Novel AlGaN/GaN High Electron-Mobility Transistor with a P-GaN Buried Layer and a Locally Doped Barrier Layer

**DOI:** 10.3390/mi15091158

**Published:** 2024-09-16

**Authors:** Juan Xiong, Xintong Xie, Jie Wei, Shuxiang Sun, Xiaorong Luo

**Affiliations:** 1 Henan Key Laboratory of Smart Lighting, School of Information Engineering, Huanghuai University, Zhumadian 463000, China; 2 State Key Laboratory of Electronic Thin Films and Integrated Devices, University of Electronic Science and Technology of China, Chengdu 610054, China; 3 College of Microelectronics, Chengdu University of Information Technology, Chengdu 610225, China

**Keywords:** GaN HEMT, single event transient (SET) effect, P-GaN buried layer, locally doped barrier layer

## Abstract

In this paper, a novel AlGaN/GaN HEMT structure with a P-GaN buried layer in the buffer layer and a locally doped barrier layer under the gate (PN-HEMT) is proposed to enhance its resistance to single event transient (SET) effects while also overcoming the degradation of other characteristics. The device operation mechanism and characteristics are investigated by TCAD simulation. The results show that the peak electric field and impact ionization at the gate edges are reduced in the PN-HEMT due to the introduced P-GaN buried layer in the buffer layer. This leads to a decrease in the peak drain current (*I*_peak_) induced by the SET effect and an improvement in the breakdown voltage (BV). Additionally, the locally doped barrier layer provides extra electrons to the channel, resulting in higher saturated drain current (*I*_D,sat_) and maximum transconductance (*g*_max_). The *I*_peak_ of the PN-HEMT (1.37 A/mm) is 71.8% lower than that of the conventional AlGaN/GaN HEMT (C-HEMT) (4.85 A/mm) at 0.6 pC/µm. Simultaneously, *I*_D,sat_ and BV are increased by 21.2% and 63.9%, respectively. Therefore, the PN-HEMT enhances the hardened SET effect of the device without sacrificing other key characteristics of the AlGaN/GaN HEMT.

## 1. Introduction

In recent years, high electron-mobility transistors (HEMTs) based on GaN/AlGaN heterostructures have made significant progress due to their excellent material properties, including high electron mobility, a high electric field strength, a wide bandgap, and more [1,2,3,4]. With the continuous improvement of microelectronics fabrication techniques, the current gain cutoff frequency (*f*_T_) and maximum oscillation frequency (*f*_max_) of GaN HEMTs have greatly increased [5,6,7], making them highly suitable for aerospace and satellite power applications [8,9,10].

When GaN HEMTs are used in space equipment, their operating characteristics can be limited by irradiation effects. One of the most common radiation effects is the single event transient (SET) effect caused by high-energy heavy ions in space [11,12,13], which can alter the operating state of the device and even lead to permanent damage. To date, the SET effects in GaN HEMTs have been extensively studied by many researchers [14,15,16,17]. The high impact ionization rate in the high electric field region of GaN HEMTs results in the generation of more electron–hole pairs, leading to a significant increase in electron collection by the drain electrode and, consequently, increased sensitivity to SET effects [18,19]. Therefore, one method to improve the radiation hardness of GaN HEMTs against SET effects is to reduce the electric field. To modulate the electric field distribution, a gate field plate is commonly utilized [20,21,22]. However, the field plate will induce additional parasitic gate capacitance, decaying the *f*_T_ and *f*_max_ of the device. Introducing a P-type buried layer structure is an effective method to modulate the channel electric field and has been reported by many researchers [23,24,25,26]. A dual-channel P-type buried layer has been used to decrease the electric field near the drain channel, resulting in an increase in single-event burnout voltage for GaN MISFETs [27]. However, the P-type buried layer reduces the electron concentration in the channel, leading to the degradation of GaN HEMT characteristics. Therefore, a method that reduces the sensitivity of the device to SET effects without sacrificing other characteristics is needed.

In this work, to enhance the SET hardening and DC characteristics of GaN HEMTs, a novel HEMT with a p-GaN buried layer in the buffer layer and a locally doped barrier layer under the gate (PN-HEMT) is proposed and investigated by TCAD simulation. It was observed that the peak drain current (*I*_peak_) induced by the SET effect in the PN-HEMT is significantly decreased due to the P-GaN buried layer. The *I*_peak_ of the PN-HEMT is 71.8% lower than that of the conventional HEMT (C-HEMT). Furthermore, it was found that the saturated drain current (*I*_D,sat_) of the PN-HEMT is slightly increased by 21.2% compared with that of the C-HEMT, due to the locally doped barrier layer.

## 2. Device Structure and Simulation Details

**Figure 1:**
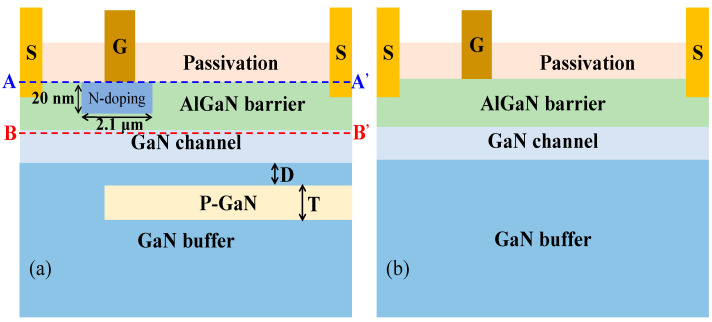
Schematic cross-section of (**a**) PN-HEMT and (**b**) C-HEMT.

Figure 1a shows the structure of the PN-HEMT. A P-GaN buried layer in the buffer layer and a locally doped barrier layer under the gate are the notable features of the PN-HEMT. The simulations are carried out in Sentaurus TCAD [28], and physics models are introduced, such as the DopingDep and High-field dependent mobility model, the piezoelectric polarization (strain) model, the impact ionization model, and the Schockley–Read–Hall recombination model. The length and thickness of locally doped Al_0.3_Ga_0.7_N barrier are 2.1 µm and 20 nm, respectively. The doping concentration of the locally doped Al_0.3_Ga_0.7_N barrier is 1 × 10^18^ cm^−3^. The distance from the P-GaN buried layer to the GaN channel (*D*) is 50 nm and the thickness of the P-GaN buried layer is 0.1 µm. The doping concentration of the P-GaN buried layer is 7 × 10^17^ cm^−3^. Figure 1b shows the structure of the C-HEMT. The work function of gate is set as 5.2 eV to model the Ni/Au Schottky contact of the actual device [29]. The other parameters are shown in Table 1.

To investigate the SET performance of the devices, the HeavyIon model is adopted. Under the harshest conditions, the incidence position of the particle is set at the gate edge closest to the drain [19,26], with the particle traveling vertically across the device. After the particle strike, the generation rate of electron–hole pairs is described by a spatial and temporal Gaussian function, which is expressed as follows [30,31]:

(1) $$rate(x,t)=\frac{LET}{q\pi \omega_{0}T_{C}}\exp\left[-\frac{{\left(x-x_{0}\right)}^{2}}{{\omega_{0}}^{2}}\right]\cdot \exp\left[-\frac{{\left(t-T_{0}\right)}^{2}}{{T_{C}}^{2}}\right]$$

where the spatial Gaussian function width *ω*_0_ and the temporal Gaussian function width *T*_C_ are set as 0.06 μm and 5 × 10^−12^ s, respectively. The initial time *T*_0_ of the charge generation is set to 2 × 10^−11^ s. The LET value in simulation is 0.6 pC/μm, which corresponds to 63.8 MeV·cm^2^/mg for Ta [32], with a conversion factor of 0.0095 [33].

## 3. Results and Discussion

### 3.1. Basic Characteristics

**Figure 2:**
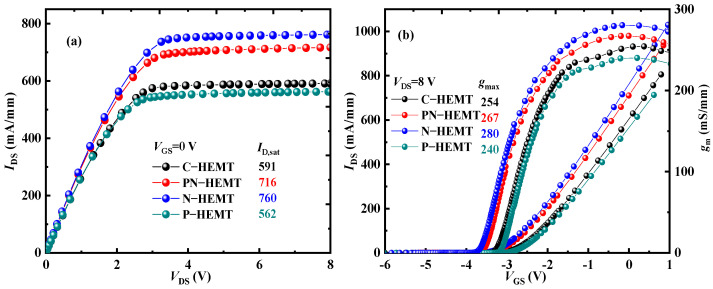
(**a**) Output and (**b**) transfer characteristics for different devices.

**Figure 3:**
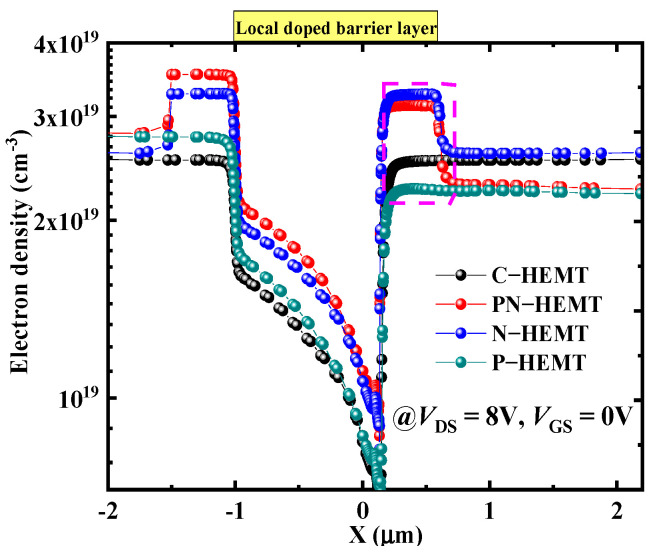
Electron concentration along the channel for different devices.

Figure 2 illustrates the DC characteristics of the PN-HEMT, C-HEMT, N-HEMT (with only the locally doped barrier layer), and P-HEMT (with only the P-GaN buried layer). The results show that a much higher saturated drain current (*I*_D,sat_) and maximum transconductance (*g*_max_) are achieved for the N-HEMT and PN-HEMT. This improvement is attributed to the locally doped barrier layer in the proposed structures. In the PN-HEMT and N-HEMT, the locally doped barrier layer provides additional electrons to the channel, thereby enhancing electron density from the *x*-coordinate at the gate’s right-side edge to the locally doped barrier’s right-side edge [34], as shown in the dashed pink box in Figure 3. Consequently, a much higher *I*_D,sat_ is observed for the N-HEMT and PN-HEMT. Moreover, the lowest *I*_D,sat_ is observed in the P-HEMT, as the buried P-GaN island partially depletes the 2DEG. Compared to the *I*_D,sat_ and *g*_max_ of 591 mA/mm and 254 mS/mm in the C-HEMT, a higher *I*_D,sat_ of 716 mA/mm and *g*_max_ of 267 mS/mm are achieved in the PN-HEMT.

**Figure 4:**
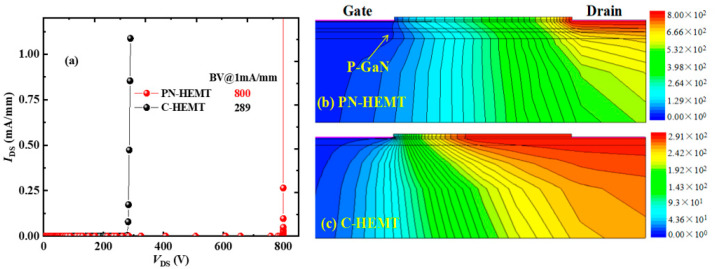
(**a**) I–V characteristics curves and (**b**,**c**) distribution of equipotential lines at breakdown.

Figure 4a compares the I−V characteristics of the PN-HEMT and C-HEMT. The breakdown voltage (BV) is extracted from the *I*_DS_−*V*_DS_ curve when *I*_DS_ = 1 mA/mm. Compared to the BV of 289 V in the C-HEMT, a higher BV of 800 V is achieved by the proposed PN-HEMT. Figure 4b,c show the distribution of equipotential lines for the PN-HEMT and C-HEMT at breakdown. As shown in Figure 4b, the equipotential lines are more uniformly distributed between the gate and drain owing to the redistribution of the electric field of the P−GaN buried layer [35,36]. However, the equipotential lines for the C-HEMT are more crowded near the gate. Additionally, the buffer leakage current is reduced by the P−GaN buried layer, further increasing the BV. Consequently, the PN-HEMT achieves a higher BV.

### 3.2. SET Effect

**Figure 5:**
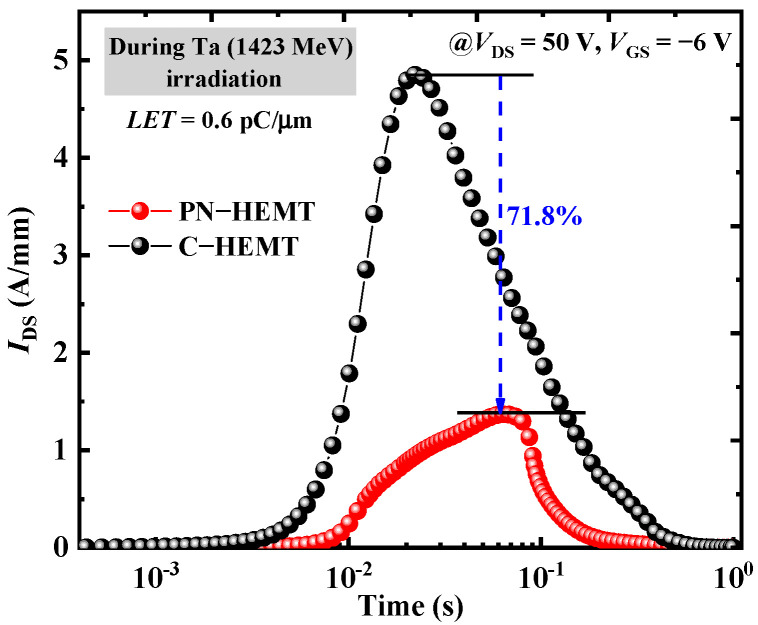
Drain currents as a function of time after heavy ion strike (*V*_DS_ = 50 V and *V*_GS_ = −6 V).

The variations in *I*_DS_ over time for the PN-HEMT and C-HEMT after a particle strike at *V*_DS_ = 50 V and *V*_GS_ = −6 V (off state) are shown in Figure 5. After the particle strike, the *I*_DS_ for both devices initially increase rapidly and reach their peaks (*I*_peak_), then quickly decrease. The *I*_peak_ of the PN-HEMT (1.37 A/mm) is 71.8% lower than that of the C-HEMT (4.85 A/mm). Additionally, the drain current pulse duration of the PN-HEMT is shorter than that of the C-HEMT. Therefore, the PN-HEMT demonstrates a much better resistance to the SET effect compared to the C-HEMT.

**Figure 6:**
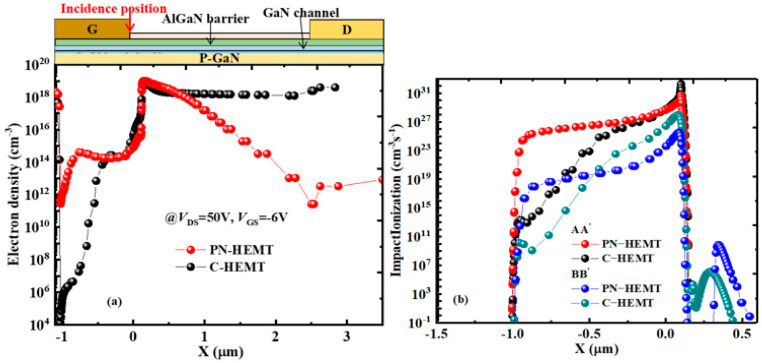
(**a**) Channel electron density distribution (along the line BB’) and (**b**) impact ionization rate for PN-HEMT and C-HEMT at peak time.

**Figure 7:**
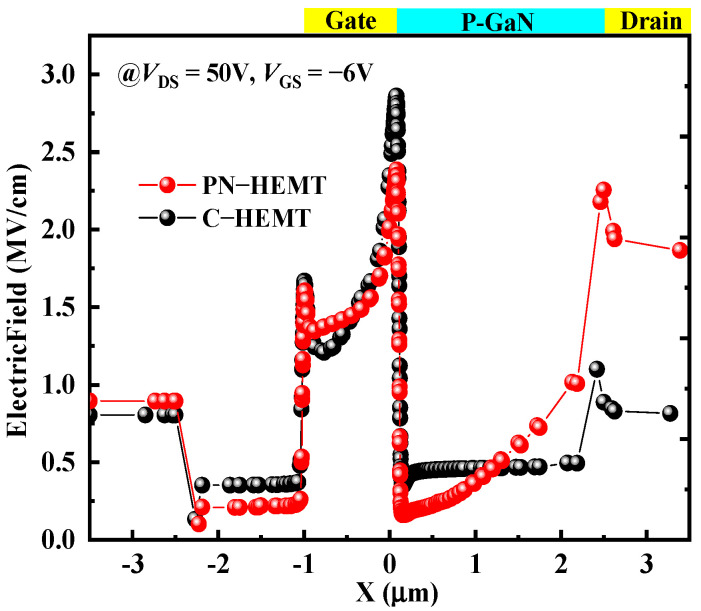
Electric field distribution (along the line BB’) at peak time.

To explain the lower *I*_peak_ for the PN-HEMT, the electron density in the channel (BB’) for the PN-HEMT and C-HEMT at peak time is analyzed, as shown in Figure 6a. It can be seen that, due to the P-GaN buried layer depleting electrons in the channel between the gate and drain in the PN-HEMT, there is a noticeable reduction in electron density. In addition, the effect on electron concentration from the locally doped barrier layer under the gate is minimal. However, the electron density remains high in the C-HEMT. To further elucidate the low electron concentration in the PN-HEMT, the impact ionization rate (IR) for the PN-HEMT and C-HEMT at peak time is analyzed, as shown in Figure 6b. The results show that the IR at the AlGaN barrier (AA’) and GaN channel (BB’) interface for the PN-HEMT is smaller than that of the C-HEMT. This is mainly due to the lower electric field along the particle incident path in the PN-HEMT, as shown in Figure 7, which suppresses electron–hole pair ionization. Hence, fewer electrons are generated in the PN-HEMT at peak time, resulting in a significantly lower *I*_peak_.

**Figure 8:**
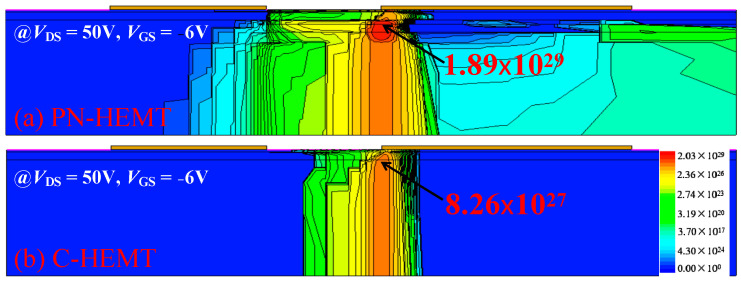
SRH recombination rate at peak time for (**a**) PN-HEMT and (**b**) C-HEMT.

In addition, the P-GaN buried layer increases the SRH recombination rate, as shown in Figure 8, resulting in more electrons being recombined before they are collected by the drain electrode. Consequently, the *I*_peak_ of the PN-HEMT is further decreased.

**Figure 9:**
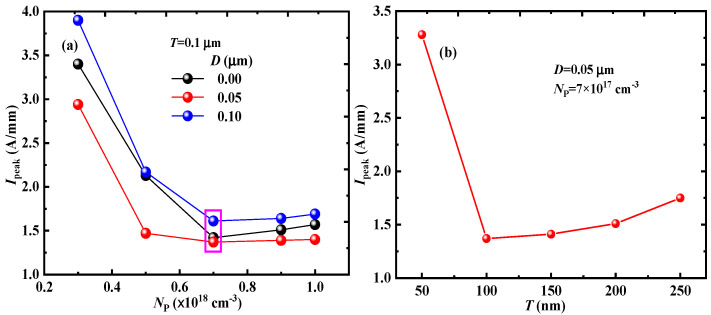
(**a**) Optimized *D* and *N*_p_ and (**b**) *T* and corresponding *I*_peak_.

**Figure 10:**
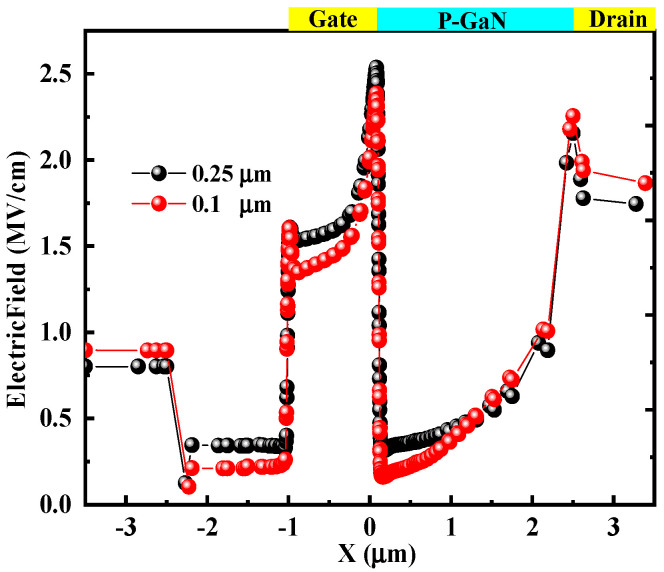
Electric field distribution for different *T* at peak time.

**Figure 11:**
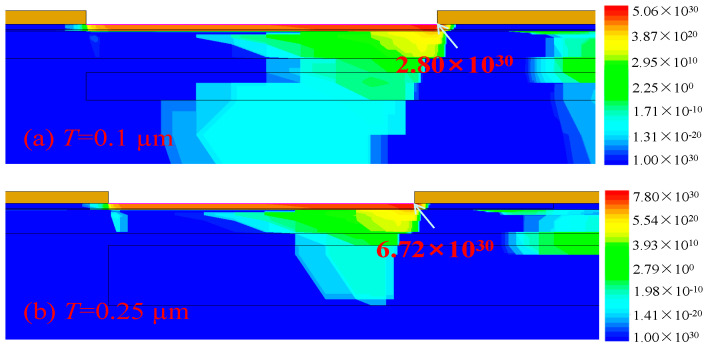
Impact ionization rate for PN-HEMT with different *T* at peak time.

The influences of *D* and *N*_P_ on the *I*_peak_ of the PN-HEMT at *T* = 0.05 μm are shown in Figure 9a. The results indicate that the *I*_peak_ of the PN-HEMT decreases to a minimum and then increases again with the increase in *D* and *N*_P_. Figure 9b shows the effects of *T* on the *I*_peak_. As *T* increases, the *I*_peak_ initially decreases and then increases. This is mainly due to the higher electric field that is obtained with the thicker *T*, as shown in Figure 10. Changes in the electric field in the device have an important effect on the IR. Therefore, the IR is higher for the thicker *T*, as shown in Figure 11, and therefore more electron–hole pairs will be generated at a thicker T, resulting in a higher *I*_peak_. When *N*_P_ is 7 × 10^17^ cm^−3^, *T* is 0.1 μm, and D is 0.05 μm, the *I*_peak_ of the PN-HEMT reaches its lowest value (1.37 A/mm). Simultaneously, *I*_D,sat_ and BV are increased by 21.2% and 63.9%, respectively. Therefore, the PN-HEMT enhances the device’s resistance to SET effects without sacrificing other key characteristics of the GaN HEMT.

**Figure 12:**
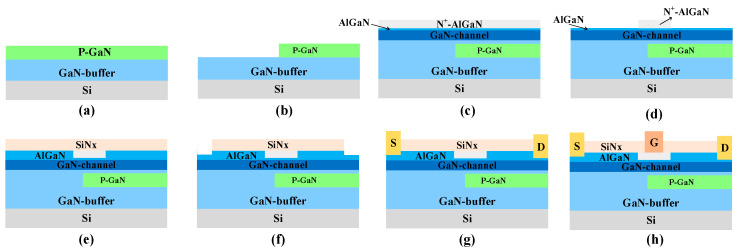
Key fabrication process steps for PN-HEMT.

Figure 12 illustrates the key feasible fabrication process flows for the PN-HEMT. The steps start with epitaxially growing GaN buffer and P-GaN layers on a Si substrate by MOCVD in Figure 12a. The realization of an epitaxial P-GaN layer could be achieved by using Mg as dopant. The P-GaN layer is selectively etched by ICP until the GaN buffer is exposed, as shown in Figure 12b, and, then, surface treatment is used to improve the surface quality [37]. The GaN buffer/GaN channel/AlGaN/N^+^-AlGaN are regrown by MOCVD, as shown in Figure 12c [38]. The ICP is utilized to selectively etch N+-AlGaN until the AlGaN layer is exposed, as shown in Figure 12d, which is followed by surface treatment. Subsequently, the regrowth of the AlGaN layer is achieved by MOCVD and a SiN_x_ passivation layer is formed by LPCVD, as shown in Figure 12e. Afterwards, digital etching is used to form the source and drain trenches, as shown in Figure 12f. The Ti/Al/Ni/Au stack is deposited with a low-temperature Ohmic process and lift-off for source and drain electrode are performed, as shown in Figure 12g. Finally, the gate electrode is formed by e-beam evaporation after selectively removing SiN_x_ by RIE, and is then lifted off, as shown in Figure 12h.

## 4. Conclusions

In this paper, a novel HEMT with a P-GaN buried layer in the buffer layer and a locally doped barrier layer under the gate is proposed to enhance resistance to SET effects. The results show that the *I*_peak_ of the PN-HEMT is significantly decreased due to the reduction in the *E*_peak_ and IR by the P-GaN buried layer, while the BV is also improved. In addition, the locally doped barrier layer provides extra electrons to the channel, enhancing electron density, resulting in a much higher *I*_D,sat_ for the PN-HEMT. Consequently, compared to the *I*_peak_ of 4.85 A/mm in the C-HEMT, the novel PN-HEMT achieves an *I*_peak_ of 1.37 A/mm, a reduction of 71.8%. The *I*_D,sat_ and BV of the PN-HEMT are increased to 716 mA/mm and 800 V, respectively, from 591 mA/mm and 289 V in the C-HEMT, representing increases of 21.2% and 63.9%, respectively.

## Figures and Tables

**Table 1:** Parameters of the PN-HEMT in simulation.

Parameter	Value
Al_0.3_Ga_0.7_N barrier layer thickness	25 nm
GaN channel layer thickness	100 nm
Thickness of P-GaN buried layer (*T*)	100 nm
Distance from channel for P-GaN buried layer (*D*)	50 nm
P-GaN layer doping concentration (*N*_P_)	7 × 10^17^ cm^−3^
GaN buffer layer thickness	1.4 µm
Gate–source spacing	1.4 µm
Gate–drain spacing	2.4 µm

## Data Availability

Data are contained within the article.
